# Exploring Variations in Median Nerve Formation: Embryological Basis and a Pathway to Improved Clinical Outcomes

**DOI:** 10.7759/cureus.106440

**Published:** 2026-04-04

**Authors:** Sanghmithra Venkateswar, Kalpana Ramachandran

**Affiliations:** 1 Anatomy, Sri Ramachandra Institute of Higher Education and Research, Chennai, IND

**Keywords:** absent musculocutaneous nerve, axillary artery, brachial plexus, median nerve, root variation

## Abstract

Aim

This study investigated the gross anatomical variability in the formation of the median nerve and evaluated its clinical and surgical implications. The research aimed to observe variations in median nerve root formation, compare differences between sides and genders, and to understand the embryological basis of these variations.

Materials and methods

A cadaveric investigation was conducted on 60 upper limbs (ULs) from 30 embalmed adult cadavers (15 males and 15 females). An infraclavicular approach was used for dissection, following standard dissection protocols. The number of roots forming the median nerve, along with their relationship to the axillary artery, was documented and analysed. Statistical analysis included chi-square and Fisher’s exact tests to correlate findings with age, gender, and laterality. A p-value of less than 0.05 was considered statistically significant.

Results

Significant variations in median nerve formation were observed in 21 (35%) of specimens. The classic two‑root pattern was present in 39 (65%) of limbs. Three‑root contributions occurred in 17 (28.33%) of cases, predominantly in males. Four‑root contributions were identified in three (5%) of specimens, while single‑root formation was observed in one (1.67%). Only one (1.67%) specimen demonstrated an absent musculocutaneous nerve, with compensatory median nerve innervation to the anterior arm muscles. Gender showed a statistically significant association with root variations, with males exhibiting a higher incidence of contributions from multiple roots.

Conclusion

Variations in median nerve formation are relatively common and clinically significant. The diversity of morphological patterns necessitates heightened awareness among surgeons, anaesthesiologists, and neurologists. Understanding these anatomical variations is essential for preventing iatrogenic injuries during surgical procedures, ensuring successful regional anaesthesia, and for accurately interpreting atypical neurological presentations.

## Introduction

The ventral rami of the lower four cervical and first thoracic spinal nerves (C5-T1) create the brachial plexus, which innervates the upper limb (UL). It is organised into roots, trunks, divisions, and cords. The fourth cervical ventral ramus may branch into the fifth as the prefixed brachial plexus, while the first thoracic ventral ramus frequently receives fibres from the second thoracic ventral ramus as the postfixed brachial plexus. The upper trunk is formed by the ventral rami of C5 and C6, the middle trunk by C7, and the lower trunk by C8 and T1. All three trunks lie immediately superior or posterior to the clavicle and split into anterior and posterior divisions. The posterior cord is the union of the posterior divisions of all three trunks. The anterior divisions of the upper and middle trunks join to form the lateral cord. The medial cord is a direct extension of the anterior division of the lower trunk [1].

The brachial plexus’ medial and lateral cords both contribute to the formation of the median nerve by giving two roots. The median nerve’s lateral root is situated lateral to the upper portion of the third segment of the axillary artery, its medial root is situated medial to the upper portion of the third segment of the axillary artery, and its trunk is located lateral to the lower portion of the third segment of the axillary artery [2]. The median nerve is lateral to the brachial artery in the upper third of the arm, crosses the artery anteriorly at the site of coracobrachialis insertion in the middle third of the arm, and then lies medially in the lower third of the arm [3].

The median nerve courses without innervating any muscles in the arm; however, it does supply sympathetic postganglionic fibres to the axillary and brachial arteries. It supplies the majority of flexor muscles in the ventral compartment of the forearm, as well as the thenar muscles and the first and second lumbricals in the hand. It also supplies the lateral aspect of the skin of the palm and the lateral two and a half fingers [2]. Fibres in the lateral root supply sensation to the palmar skin of the thumb, index, and part of the middle finger, as well as to the pronator teres, flexor carpi radialis, and half of the flexor digitorum superficialis [3]. The lateral root, arising from the lateral cord, carries fibres from the C5, C6, and C7 spinal nerves, while the medial cord, from which the medial root arises, transmits fibres from the C8 and T1 spinal nerves [4].

Variations in UL nerve anatomy are critical considerations during surgical procedures, particularly extensive neck surgeries, where unrecognised differences may increase the risk of injury. They are also important considerations when assessing atypical nerve entrapment symptoms [1]. The greatest variance linked to median nerve formation occurs in the lateral cord, where the lateral root of the median nerve can be formed by two or three heads [5].

Structural variability in the median nerve has clinical significance for anaesthesiologists as well as surgeons. Being aware of these morphological variations can help prevent iatrogenic damage during surgical and anaesthetic procedures around the axilla [5]. This article describes the structural variations of the median nerve, its developmental perspectives, and contributions to existing knowledge, highlighting its structural and clinical importance [4].

Aim and objectives

The aim of this study is to investigate the gross anatomical variations of the median nerve, as well as its clinical and surgical implications. The objectives of this study are: (1) to observe changes in median nerve root formation, and (2) to compare differences in median nerve formation on both sides and across genders.

## Materials and methods

This cadaveric investigation was conducted in the Department of Anatomy of a tertiary care hospital affiliated with a medical college. Institutional Ethical Committee (IEC) clearance was obtained (IEC no. CSP-MED/24/SEP/109/321). The study used 60 ULs from 30 embalmed adult cadavers, comprising 15 female and 15 male cadavers. Sample size estimation was performed by consecutive sampling (i.e., by including all available samples that met the inclusion criteria during the one-year study period).

Both adult and geriatric cadavers with intact axillary regions and no previous dissections were included. Cadavers showing signs of decomposition, trauma, surgical procedures, or pathological or infectious changes in the axillary region were excluded. All cadavers had their ULs dissected in accordance with Cunningham's Manual of Practical Anatomy [4]. 

An infraclavicular approach was used. The cadaver was positioned supine, with the arm fully abducted and laterally rotated. In the axillary region, a curved oblique skin incision of approximately 7 cm was made along the axillary fold, extending from the lateral border of the pectoralis major to the anterior border of the latissimus dorsi, using these structures as anatomical landmarks. The curvature of the incision followed the natural contour of the axillary fold to allow optimal exposure.

The skin flaps were carefully reflected in the subcutaneous plane, preserving the superficial fascia. Dissection was then continued in the plane superficial to the deep (axillary) fascia, ensuring clear exposure of the underlying neurovascular structures while maintaining anatomical integrity.

The pectoralis major muscle was identified by its attachment to the clavicle, sternum, and humerus. The muscle was carefully retracted superolaterally using blunt dissection to expose the underlying pectoralis minor and deeper axillary contents. Then, the clavipectoral fascia was carefully dissected, and the pectoralis minor was cut from its origin in the ribs and reflected superiorly toward its attachment on the coracoid process. The axillary fat pad and lymph nodes, if present, were next dissected to reveal the axillary artery and brachial plexus cords (medial, lateral, and posterior), which were identified in association with the second part of the axillary artery.

The cords were traced distally, and the major terminal branches were identified. The musculocutaneous nerve was identified by tracing the lateral cord, which pierced and ran through the coracobrachialis muscle. The median nerve was identified superficial and medial to the lower half of the third segment of the axillary artery. The number of roots forming the median nerve, as well as the cadaver's gender and side, were all noted. Photos were captured and tagged. The acquired data were charted and compared with existing literature.

The number of specimens exhibiting variations in the formation of the median nerve was expressed as frequencies and percentages. Associations between anatomical variations and categorical variables, such as gender and laterality, were analysed using the chi-square test and Fisher’s exact test.

A p-value was calculated for each comparison, and a value of < 0.05 was considered statistically significant. Statistical analysis was performed using IBM SPSS Statistics for Windows, Version 23 (Released 2015; IBM Corp., Armonk, NY, USA).

## Results

Distribution of age and gender among the cadavers

An equal number of male and female cadavers were observed, with 15 (50.0%) in each group. Table 1 illustrates the age distribution of the cadavers studied.

**Table 1:** Frequency distribution of age among the cadavers (n = 30) Data are represented as n (%).

Age group	No. of cadavers
41 - 50	1 (3.4%)
51 - 60	11 (36.6%)
61 - 70	10 (33.4%)
71 - 80	8 (26.6%)

Variations in median nerve root formation

**Figure 1:**
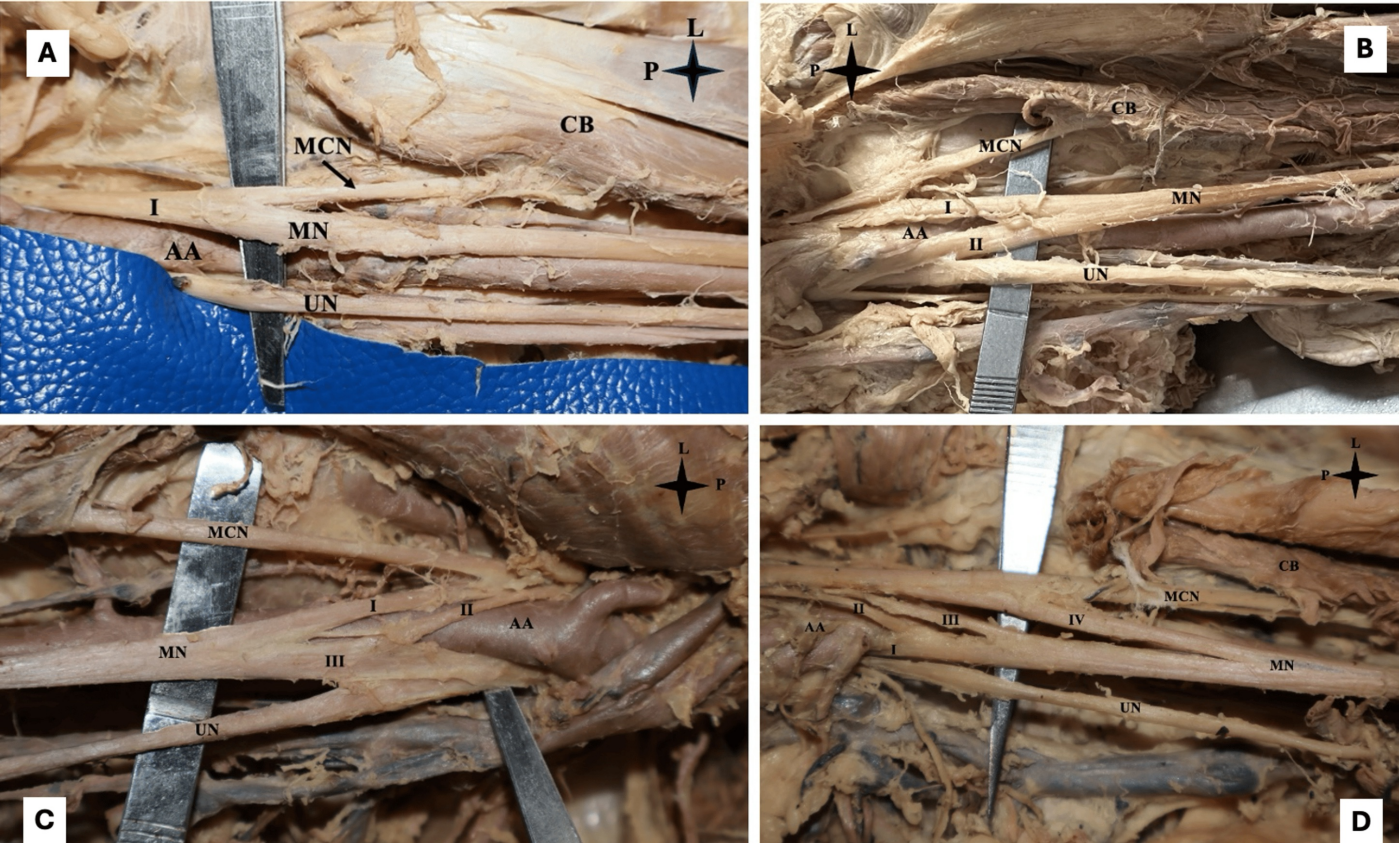
Variation in the formation of median nerve A) Left arm showing the median nerve with a single root arising from the lateral cord of the brachial plexus; B) Left arm showing the median nerve with two roots, one root arising from the lateral cord of the brachial plexus and one root arising from the medial cord of the brachial plexus; C) Right arm showing the median nerve with three roots, two roots arising from the lateral cord of the brachial plexus and one root arising from the medial cord of the brachial plexus; D) Left arm showing the median nerve with four roots, three roots arising from the lateral cord of the brachial plexus and one root arising from the medial cord of the brachial plexus. I, II, III, IV - roots of MN. L: lateral side; P: proximal; MN: median nerve; MCN: musculocutaneous nerve; UN: ulnar nerve; AA: axillary artery; CB: coracobrachialis

The variations in the formation of the median nerve are shown in Table 2. Only one (1.67%) of the 60 ULs had a median nerve with single-root development (Figure 1A). A male cadaver’s left UL displayed this variation. In this arm, a single root emerged from the lateral cord of the brachial plexus, but the root that normally originates from the medial cord of the brachial plexus was absent. The lateral root was lateral to the upper half of the third segment of the axillary artery and continued as the median nerve lateral to the lower portion of the third segment of the axillary artery. 

**Table 2:** Variation in the formation of median nerve Median nerve variations among cadavers. Data are presented as n (%).

No. of roots	Male limbs (n = 30)	Female limbs (n = 30)	Total no. of limbs (n = 60)
1 root	1 (3.33%)	0 (0%)	1 (1.67%)
2 roots	13 (43.33%)	26 (86.67%)	39 (65%)
3 roots	14 (46.67%)	3 (10%)	17 (28.33%)
4 roots	2 (6.67%)	1 (3.33%)	3 (5%)

Out of the 60 UL, 39 (65%) had two roots of the median nerve (Figure 1B). This structure was observed in five (12.8%) right ULs of male cadavers and eight (20.5%) left ULs of male cadavers, as well as 12 (30.8%) right ULs and 14 (35.9%) left ULs of female cadavers. In these limbs, one root came from the lateral cord, and the other arose from the medial cord in relation to the upper section of the third segment of the axillary artery. In all these limbs, the root from the medial cord crossed the third segment of the axillary artery anteriorly toward the lateral side to link with the root originating from the lateral cord. Thus, the median nerve, which formed in the lower half of the third part of the axillary artery, was lateral to it.

**Figure 2:**
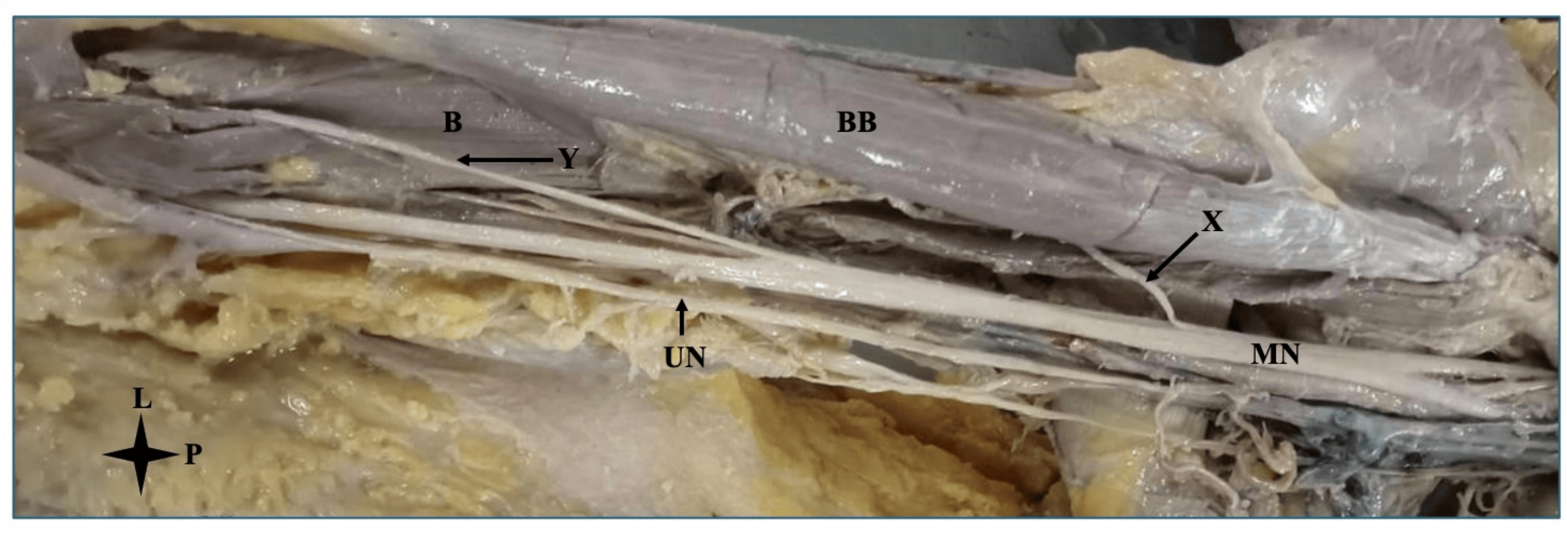
Median nerve supplying muscles in the arm The right arm showing absent MCN. MCN: musculocutaneous nerve; L: lateral; P: proximal; MN: median nerve; UN: ulnar nerve; BB: biceps brachii; X: branch from MN supplying BB; B: Brachialis; Y: branch from MN supplying B

In one (1.67%) of the cadavers (male, right side), the musculocutaneous nerve was absent, but the median nerve was formed by two roots, one from each cord (medial and lateral) (Figure 2). The medial root crossed the third segment of the axillary artery anteriorly to the lateral side. As a result, the formed median nerve was lateral to the third segment of the axillary artery. Branches of the median nerve supplied the coracobrachialis, biceps brachii, and brachialis.

Out of the 60 ULs, 17 (28.33%) had three-root formation of the median nerve (Figure 1C). This variant was observed in eight (47.06%) right ULs of male cadavers, six (35.29%) left ULs of male cadavers, and in two (11.76%) right ULs and one (5.88%) left UL of female cadavers. In these limbs, two roots emerged from the lateral cord and one from the medial cord, in relation to the upper half of the third segment of the axillary artery. In all these limbs, the two roots of the lateral cord crossed the third segment of the axillary artery anteriorly, towards the medial side, to unite with the single root originating from the medial cord. Thus, the median nerve formed was ventromedial to the lower section of the third segment of the axillary artery.

Only three (5%) of the 60 ULs demonstrated four-root formation of the median nerve (Figure 1D). This variant was found in the right ULs of two (66.67%) male and one (33.33%) female cadavers. In these limbs, three roots originated from the lateral cord and one from the medial cord, in relation to the upper portion of the third part of the axillary artery. In all of these limbs, the three roots from the lateral cord crossed the axillary artery anteriorly, to the medial side, where they joined the single root from the medial cord. Thus, the median nerve formed was anteromedial to the lower section of the third segment of the axillary artery.

Using Fisher's exact test, and based on Table 3, we can infer the relationship between gender and the number of median nerve roots on both sides. The median nerve was most frequently formed by two roots in both sexes, with a higher frequency among females, 26 (66.67%), compared to males, 13 (33.33%). Three-root formations were predominantly observed among males (14, 82.35%) compared to females (3, 17.65%), while four-root formations were seen in two (66.67%) males and one (33.33%) female. A single-root formation was observed only in one male (100%). The association between gender and the number of roots on both sides was statistically significant (χ² = 12.784, p = 0.0006).

**Table 3:** Correlation between the number of roots of the median nerve (both sides) and gender Correlation of the number of roots of the median nerve (both sides) with gender. Data are presented as n (%). Statistical significance was defined as p < 0.05. * denotes Fisher’s exact test.

Number of roots (both sides)	Gender		p-value (Chi-square value)*
	Male	Female	
One root (n = 1)	1 (100.00%)	0 (0.0%)	0.0006 (12.784)
Two roots (n = 39)	13 (33.33%)	26 (66.67%)	
Three roots (n = 17)	14 (82.35%)	3 (17.65%)	
Four roots (n = 3)	2 (66.67%)	1 (33.34%)	

From Table 4, we can infer the correlation between the number of roots of the median nerve on both sides and the right and left sides using Fisher’s exact test. The most common pattern observed was the presence of two roots, seen in 39 (65%) specimens - 17 (43.58%) on the right and 22 (56.42%) on the left side. Three-root formations were noted in 17 (28.33%) specimens, more commonly on the right side (10, 58.82%) than on the left (7, 41.18%). A single-root formation was observed only on the left side, while all four-root formations were present exclusively on the right side. The association between the number of roots and side was not statistically significant (χ² = 5.382, p = 0.099).

**Table 4:** Correlation of the number of roots of the median nerve with side (right and left) Correlation of the number of roots of the median nerve with side (right and left). Data are presented as n (%), and statistical significance was defined as p < 0.05. * denotes Fisher’s exact test.

Number of roots (both sides)	Side		p-value (Chi-square value)*
	Right	Left	
One root (n = 1)	0 (0.0%)	1 (100.00%)	0.099 (5.382)
Two roots (n = 39)	17 (43.58%)	22 (56.42%)	
Three roots (n = 17)	10 (58.82%)	7 (41.18%)	
Four roots (n = 3)	3 (100.00%)	0 (0.0%)	

## Discussion

The median nerve is made up of two roots: one from the lateral cord (C5, C6, and C7) and another from the medial cord (C8 and T1). Both roots enclose the third segment of the axillary artery and unite on its anterior or lateral side. A few fibres of the seventh cervical ventral ramus originate from the lateral root in the lower axilla. They pass distal and medial to the medial root, often ventral to the axillary artery, before connecting to the ulnar nerve. Clinically, the median nerve is thought to have a primary motor function with a small sensory component. The median nerve also gives rise to vascular branches that supply the brachial artery. If the lateral root is small, the median nerve is joined by the musculocutaneous nerve (C5, C6, and C7) in the arm [3].

Embryological basis

The anatomical variations of the median nerve can be explained using embryological development principles. The embryological basis of UL innervation includes a highly coordinated process of neuronal specification, axonal extension, and molecular guidance. This process ensures that motor and sensory axons from the spinal cord precisely reach their targets in the developing limb.

The UL bud first appears around day 24 of embryonic development [6]. The motor neurons are situated in the ventral spinal cord. The peripheral sensory and autonomic neurons, along with their associated glia, originate from neural crest cells [6,7]. Motor axons begin to enter the limb bud around the fifth week.

The identity of neurons that will innervate the limb is determined by specific genetic programs: the lateral motor column (LMC) of the spinal cord houses motor neurons that control the limbs [6,7]. At the brachial level (C5-T1), their identity is specified by Hox6-8 and the accessory factor Foxp1 [7].

The LMC is divided into two main parts based on transcription factors. Medial LMC (LMCm) neurons (expressing Isl1 and Isl2) are programmed to innervate the ventral limb compartment, while lateral LMC (LMCl) neurons (expressing Lim1 and Isl2) are programmed to innervate the dorsal limb compartment [6,7].

As axons exit the spinal cord, they migrate along permissive channels to the origin of the limb bud, where they combine and create the brachial plexus. The pathway is near the developing axis artery (seventh intersegmental artery-subclavian artery, SA) and also near the developing humerus. This also influences the division of axonal bundles. If the axis artery develops from any intersegmental artery other than the seventh, it can cause the axonal bundles to divide in an uncommon fashion, resulting in differences in the brachial plexus [8]. 

If the SA does not transect the anterior-division (AD) axon plane, the AD can merge symmetrically into a single anterior bundle, analogous to the posterior division’s normal symmetric convergence. This can cause the median nerve to appear as one common root, rather than arising from separate lateral and medial roots [8].

The SA normally runs diagonally through the AD axon plane, forming an obstacle to converging AD bundles. This forces a stepwise fusion of the C5-C6 anterior division and the C7 anterior division, which are pushed together cranial to the SA, where they meet and merge into the C5-C7 anterior divisions (lateral cord). Distal to the SA, the C5-C7 anterior divisions meet and merge with the C8-T1 anterior divisions (medial cord) to form the C5-C8, T1 anterior division, which extends as the median nerve. This is the normal formation of two roots of the median nerve, which reflect these two major converging contributors (C5-C7 anterior divisions and C8-T1 anterior divisions) [8].

Three roots of the median nerve can occur via multiple strands and/or be altered by vessels. Multi-stranded convergence occurs at the C5-C7 anterior divisions and the C8-T1 anterior divisions union. Near the SA, the C5-C7 anterior divisions can broaden and branch into many narrow axon strands before uniting with the C8-T1 anterior divisions. Thus, instead of a single lateral root, two lateral roots join the median nerve alongside the single medial root. Apart from this type, the C5-C7 anterior divisions bundle can split around the veins encountered along its path, after which it may remerge. However, a long split can make the median nerve appear as if it has extra roots or as a proximally split median nerve. A split C5-C7 anterior division or a split proximal median nerve can be interpreted grossly as additional median nerve roots [8].

A four-root median nerve is formed when more than two AD contributors remain separate until the very distal end. If merging of C5, C6, and/or C8-T1 is delayed, brachial division can materialise before those trunk-level fusions, i.e., the division happens proximal to normal trunk formation. This results in multiple separate AD bundles that later join to form the median nerve, rather than one consolidated lateral contributor and one medial contributor. Also, when the SA is effectively positioned such that C8 and T1 do not merge proximally (eighth intersegmental artery), the C8 anterior division and T1 anterior division grow out on opposite sides of the SA (eighth intersegmental artery) and merge more distally. These separate C8-derived and T1-derived contributions can persist as two distinct roots, which, combined with separated contributions on the lateral side, result in an apparent four-root median nerve [8].

Thus, the root number is related to AD axon bundle fusion (C5, C6 anterior divisions; C7 anterior division; C8-T1 anterior divisions), influenced by the SA course and vascular obstacles that promote splitting and multi-stranded fusion.

Simultaneously, the limb mesenchyme matures and establishes the necessary molecular cues for further guidance. Signals such as retinoic acid, ephrins, and semaphorin 3A regulate the axons leaving the plexus [6,7].

Axons reach their targets using growth cones, which are sensory-motor organs at the axonal tip that respond to environmental cues [9]. These cues can be chemoattractants or chemorepellents [4,9].

At the base of the limb, LMCl axons utilise the receptor EphA4 to detect and avoid ephrin-A ligands located in the ventral limb mesenchyme, effectively pushing them into the dorsal compartment, whereas LMCm axons utilise EphB receptors to avoid ephrin-B in the dorsal limb, and some use neuropilin-2 to avoid semaphorin 3F expressed dorsally. If there are any alterations in these chemoreceptors, they can lead to variations in the brachial plexus. Once axons reach their general muscle compartments, local cues from the muscles themselves regulate final branching and synaptogenesis. Generally, axons from the dorsal divisions of the plexus innervate extensors and supinators, while those from the ventral divisions innervate flexors and pronators. Between the sixth and eighth weeks, the UL rotates laterally. This rotation influences the final dermatomal and muscular innervation patterns [6,7].

Comparison with other studies

Sharma's study dissected 30 UL specimens from 15 male cadavers and found median nerve anomalies in 16.66% of cases [10]. Our study of the same number showed a significantly higher variation, at 56.67% (17 limbs), with three-root formation most common at 46.67%, four-root formation in 6.67%, and single-root formation in 3.33%.

Encarnacion et al.'s study team examined 84 brachial plexuses from 42 cadavers and found variations in 22.6%, more frequent in males (81.8%) than in females (18.2%). Three-root formation occurred in 20.2% and four-root formation in 2.4% [11]. Our study similarly showed male predominance (56.67% vs. 13.33% in females) and additionally documented single-root formation.

Nasr's study examined 60 ULs and found standard two-root formation in 88.3% and three-root formation in 11.7%. Musculocutaneous nerve absence occurred in 3.3% [12]. Our study found two-root formation in 65%, three-root in 28.3%, four-root in 5%, single-root in 1.67%, and absent musculocutaneous nerve in 1.67%.

Kumari et al.'s study team examined 106 ULs and found three-root formation in 26.41%, four-root formation in 1.88%, and medial positioning relative to the axillary artery in 8.49% [13]. Our study showed higher rates: three-root at 28.3%, four-root at 5%, and medial positioning at 33.3%, plus one case of single-root formation.

Patil et al.'s study dissected 68 axillae and found a standard two-root pattern in 64.7%, single-root pattern in 2.9%, three-root pattern in 27.9%, and four-root pattern in 4.4%. Extra roots originated from the musculocutaneous nerve (14.7%) or lateral cord (11.7%). Their study showed no gender significance (p > 0.05) [14]. Our study showed higher variation rates, with extra roots exclusively from the lateral cord, and significant gender differences.

Bala's case report revealed a case in which the medial root obtained an additional branch from the lateral cord, with median nerve formation occurring unusually low in the arm [15]. Our study found no supplementary branches or low-origin variations.

Ghosh et al.'s study examined 60 ULs and found variations in 30%, with three-root formation in 21.7% and four-root formation (including posterior cord contribution) in 5.0% [1]. Our study showed a higher three-root incidence (28.3%) and the same four-root rate (5%), but no posterior cord contribution. We also noted single-root formation.

Akhtar et al.'s study examined 84 ULs in North India, finding three-root formation in 23% and four-root formation in 6%, with extra roots from the lateral cord or musculocutaneous nerve. Males were more likely to exhibit variations (81.8%) [4]. Our study found extra roots only from the lateral cord, included single-root formation, and confirmed male predominance.

Passey et al.'s study examined 40 ULs and found triple-root formation in 15%, including unique “Y”-shaped and supernumerary root morphologies [5]. Our study's three-root formations from the lateral cord showed no abnormal morphologies, and we documented single- and four-root formations absent in their study.

Omuga et al.'s study dissected 70 cadavers in Western Kenya and found median nerve variations in 22 plexuses, including three-root formation, contributions from the musculocutaneous or posterior cord, and single-root formation. They found significant gender differences (p = 0.008) [16]. Our study documented one-, two-, three-, and four-root formations with no nerve communications, but confirmed significant gender differences.

Rohini and Kishve's study examined 30 arms and found complete musculocutaneous nerve absence in 6.7% (one male right arm and one female left arm), with the median nerve compensating for muscle innervation and forming the lateral cutaneous nerve of the forearm [17]. Our analysis discovered one such example in a male's right arm; however, the lateral cutaneous nerve of the forearm was missing.

Borthakur et al. reported a case showing a unique left-side variation with a lacking musculocutaneous nerve, where the lateral cord provided the coracobrachialis and the median nerve innervated the biceps and brachialis before continuing as the lateral cutaneous nerve of the forearm [18]. Our study found unilateral right-arm absence, with the median nerve supplying all anterior compartment muscles, including the coracobrachialis.

Priya et al.'s study examined 60 ULs and found a three-root median nerve in 13.33%, an absent musculocutaneous nerve in 5%, and nerve communications in 13.33% [19]. Our study showed a higher three-root prevalence, plus single- and four-root formations, one absent musculocutaneous nerve case, and no nerve communications.

Bhat et al.'s study documented rare neural loops, where the lateral and medial roots of the median nerve are wrapped around the axillary veins [20]. Our findings revealed no such looping variations, with all lateral cord roots starting normally in association with the third segment of the axillary artery. Comparison of median nerve formation across various studies is presented in Table 5.

**Table 5:** Comparison of median nerve formation between various studies

Study	Sample size	Key findings	Comparison with current study	Similarities	differences
Sharma [10]	30 limbs (15 male cadavers)	3-root: 16.66%; 2 roots from the lateral cord, 1 from the medial cord	Current Study (Male subset): 3-root: 46.67% (14/30 limbs); 4-root: 6.67% (2 limbs); 1-root: 3.33% (1 limb); Total variations: 56.67%	Both studied male cadavers with 30 limbs	3.4× higher variation rate; Additional 1-root and 4-root formations
Encarnacion et al. [11]	84 plexuses (42 cadavers)	Total variations: 22.6%; 3-root: 20.2%; 4-root: 2.4%; Males: 81.8%, Females: 18.2%; Lateral cord = common source	3-root: 28.3% (higher); 4-root: 5% (higher); Males: 56.67%, Females: 13.33%	Lateral cord as common source; Male predominance; Similar 4-root incidence	1.55× higher total variations; Additional 1-root formation; Different gender distribution ratio
Nasr [12]	60 limbs (30 cadavers)	2-root: 88.3%; 3-root: 11.7%; Absent musculocutaneous nerve (MC): 3.3%; Median nerve supplied anterior arm	2-root: 65% (lower); 3-root: 28.3% (2.4× higher); 4-root: 5% (new); 1-root: 1.67% (new); Absent MC: 1.67%(similar)	Same sample size; Absent MC with median nerve compensation; Median nerve supplies anterior arm muscles	2.4× higher 3-root formation; Lower normal anatomy rate; Additional 1-root and 4-root variants
Kumari et al. [13]	106 limbs (53 cadavers)	3-root: 26.41%; 4-root: 1.88%; Medial to axillary artery: 8.49%	3-root: 28.3% (higher); 4-root: 5% (2.7× higher); 1-root: 1.67% (new); Medial position: 33.3% (4× higher)	Similar 3-root incidence; Presence of 4-root formation	Much higher medial positioning; Higher 4-root incidence; Additional 1-root formation
Patil et al. [14]	68 axillae (34 cadavers)	2-root: 64.7%; 1-root: 2.9%; 3-root: 27.9%; 4-root: 4.4%; Extra root from MC nerve: 14.7%; Extra root from lateral cord: 11.7%; Gender: Not significant (p > 0.05)	2-root: 65% (similar); 1-root: 1.67%(lower); 3-root: 28.3% (similar); 4-root: 5% (similar); Extra roots: Only from lateral cord; Gender: Significant (p = 0.0006)	Very similar distribution of root formations; Similar 2-root percentage	Statistically significant gender difference; No MC nerve contribution; Exclusive lateral cord origin
Bala [15]	30 limbs	Supplementary branch from lateral cord; Low origin (upper/middle third of arm); Case: 45-year-old male, right limb	No supplementary branches; Normal origin level (axillary region)	-	No low origin variations; No supplementary branching
Ghosh et al. [1]	60 limbs (30 cadavers)	Total variations: 30%; 3-root: 21.7%; 4-root: 5.0%; Posterior cord contribution (novel)	Total variations: 35%(higher); 3-root: 28.3% (higher); 4-root: 5% (same); 1-root: 1.67% (new); No posterior cord contribution	Same sample size; Identical 4-root incidence; 2 roots from lateral cord	Higher overall variation rate; No posterior cord involvement; Additional 1-root formation
Akhtar et al. [4]	84 limbs (North Indian)	3-root: ~23% (M: 25%, F: 21.42%); 4-root: ~6% (M: 5.36%, F: 7.14%); Extra roots from lateral cord or MC nerve; Males: 81.8%, Females: 18.2%	3-root: 28.3% (higher); 4-root: 5%(similar); 1-root: 1.67% (new); Extra roots: Only lateral cord; Males > Females (similar trend)	Male predominance; Similar 4-root incidence; Lateral cord involvement	No MC nerve contribution; Additional 1-root formation; Exclusive lateral cord contribution
Passey et al. [5]	40 limbs (North Indian)	3-root: 15%; "Y" shaped extra root; Supernumerary root from lateral cord	3-root: 28.3% (1.9× higher); 4-root: 5%(new); 1-root: 1.67% (new)	Lateral cord as source; 3-root formation present	Higher 3-root incidence; Additional 1-root and 4-root formation
Omuga et al. [16]	70 cadavers (Western Kenya)	3-root: 6 cadavers; 1-root: Present; MC nerve or posterior cord contributions; Gender: Significant (p = 0.008)	1-root: 1 limb; 2-root: 39 limbs; 3-root: 17 limbs; 4-root: 3 limbs; No communications with other nerves; Gender: Significant (similar)	1-root formation present; Statistically significant gender difference	No nerve communication; No MC or posterior cord contributions
Rohini and Kishve [17]	30 arms (15 cadavers)	Absent MC: 6.7% (2 limbs) - 1 male right arm - 1 female left arm; Median nerve → lateral cutaneous nerve	Absent MC: 1.67% (1 limb) - 1 male right arm only; Median nerve supplied anterior compartment; Lateral cutaneous nerve absent	Absent MC in male right arm; Median nerve compensation	Lower MC absence rate; No lateral cutaneous nerve formation
Borthakur et al. [18]	1 cadaver (case report)	Left side: Absent MC; Lateral cord → coracobrachialis; Median → biceps & brachialis; Continued as lateral cutaneous nerve	Right arm: Absent MC; Median nerve→ all anterior compartment muscles (including coracobrachialis); No lateral cutaneous nerve formation	Absent MC nerve; Median nerve compensation	Different side (right vs left); Direct median nerve supply to all muscles; No lateral cutaneous continuation
Priya et al. [19]	60 limbs	3-root: 13.33%; Absent MC: 5%; Communications: 13.33%; 2 from lateral cord, 1 from medial	3-root: 28.3% (2.1× higher); 4-root: 5% (new); 1-root: 1.67% (new); Absent MC: 1.67% (lower); No communications	Same sample size; Median nerve supplies the anterior arm in case of the MC nerve absence	2× higher 3-root formation; Additional variants (1-root, 4-root); No nerve communications; Lower MC absence rate

Clinical significance

Findings of this study carry substantial implications across multiple medical specialities: neurosurgeons, orthopaedic surgeons, plastic surgeons, and healthcare providers operating in the axillary or arm region must be aware of supernumerary roots and variant median nerve courses, as failure to recognise accessory nerve bundles may result in iatrogenic injury, leading to unpredictable neurological deficits. Knowledge of these variations is essential during procedures such as trauma repair, tumour excision, nerve grafting, and transfers.

For anaesthesiologists performing brachial plexus blocks (particularly the axillary approach), variations in median nerve formation necessitate meticulous technique to ensure complete anaesthesia, awareness that multiple roots may require modified injection sites, and vigilance to avoid incomplete blocks, complications, or nerve injuries.

Neurologists and clinicians should consider anatomical variations when interpreting atypical presentations of sensory or motor loss, recognising that if the median nerve assumes musculocutaneous nerve functions (as in cases of absent musculocutaneous nerve), damage to the median nerve could cause unexpected weakness in arm flexor muscles (coracobrachialis, biceps brachii, and brachialis). Nerve conduction studies and EMG interpretations should account for possible variant innervation patterns to avoid misdiagnosis. Additionally, documentation of expected anatomical variations protects clinicians during surgical complications and ensures that informed consent adequately covers potential risks, carrying significant medicolegal relevance.

Limitations

This study's limitations include a low sample size of only 60 ULs from 30 cadavers, which may restrict the generalisability of the findings to the larger population, and regional specificity, since the cadavers were obtained from a single institution, potentially introducing regional or ethnic bias that may not reflect variations in other populations. Preservation effects may also be a concern, as formalin fixation may alter tissue properties, potentially affecting the accurate identification of fine neural structures and communications. Being a cadaveric study, there is an absence of functional correlation, as it was not possible to correlate anatomical variations with clinical symptoms or functional outcomes in living subjects. Despite bilateral comparisons being made, there is a limitation in unilateral analysis, since the study could not assess whether variations were consistently bilateral or predominantly unilateral within the same individual.

Future recommendations

Larger multicentre studies should be conducted through collaborative research across multiple institutions, with larger sample sizes to establish more accurate prevalence rates, and diverse population studies should include different ethnic groups, geographic regions, and age distributions to identify population-specific variation patterns. Clinical-anatomical correlation should integrate cadaveric findings with preoperative imaging (MRI neurography and ultrasound) and surgical observations, while functional studies should investigate whether anatomical variations correlate with specific clinical symptoms or neurological deficits. Imaging protocol development should create standardised ultrasound and MRI protocols for the preoperative identification of median nerve variations, and genetic and molecular research should explore genetic markers and developmental signalling pathways associated with brachial plexus variations. Classification system refinement should aim to develop comprehensive classification systems incorporating all observed variation patterns for standardised reporting, as the high incidence and morphological diversity of median nerve variations mandate heightened clinical awareness and the utilisation of this anatomical knowledge for safer interventions and accurate diagnosis of unexplained UL pathology.

## Conclusions

This anatomical study confirms that variations in median nerve formation are relatively common, with deviations from the typical two-root pattern occurring in a significant percentage of specimens. The most frequently observed variation was the presence of accessory roots from the lateral cord. While uncommon, mutations included single-root development and the loss of the musculocutaneous nerve; in such cases, the anterior arm compartment muscles received compensatory median nerve input.

These anatomical variations hold substantial clinical significance for surgeons, anaesthesiologists, neurologists, and health care providers. Awareness of supernumerary roots and variant nerve courses is essential to prevent iatrogenic injuries during surgical procedures and to ensure successful regional anaesthesia. Additionally, understanding these variations aids in the accurate interpretation of atypical sensory or motor deficits. In conclusion, the diverse morphological patterns of median nerve formation necessitate that clinicians maintain vigilance and incorporate this anatomical knowledge into clinical practice for safer interventions and precise diagnosis. Further studies with larger, diverse populations are recommended to better define the prevalence and clinical implications of these variants.
